# An Updated Systematic Review of *Vaccinium myrtillus* Leaves: Phytochemistry and Pharmacology

**DOI:** 10.3390/pharmaceutics15010016

**Published:** 2022-12-21

**Authors:** Ruxandra Ștefănescu, Eszter Laczkó-Zöld, Bianca-Eugenia Ősz, Camil-Eugen Vari

**Affiliations:** 1 Department of Pharmacognosy and Phytotherapy, Faculty of Pharmacy, George Emil Palade University of Medicine, Pharmacy, Science and Technology of Targu Mures, 540139 Targu Mures, Romania; 2 Department of Pharmacology and Clinical Pharmacy, Faculty of Pharmacy, George Emil Palade University of Medicine, Pharmacy, Science and Technology of Targu Mures, 540139 Targu Mures, Romania

**Keywords:** bilberry, leaves, *Vaccinium myrtillus*, phytochemistry

## Abstract

Bilberry leaves are used in many countries in traditional medicine for treating a wide variety of diseases. Due to the high therapeutic potential of *Vaccinium myrtillus* (VM) leaves, this review aims to present the latest knowledge on the phytochemical profile, as well as the therapeutic effects of this herbal drug. The review was conducted according to the Prisma guidelines, and the scientific databases were searched using combinations of the following keywords: “*Vaccinium myrtillus*”, “leaves”, “bilberry”. Recent research was focused on the influence of abiotic factors on the phytochemical composition, and it seems that there are significant differences between the herbal drugs collected from different countries. The phytochemical composition is correlated with the broad spectrum of pharmacological effects. The paper outlines the potent antimicrobial activity of VM extracts against multidrug-resistant bacterial strains, and also the pathways that are modulated by the unique “cocktail” of phytoconstituents in different metabolic alterations. Reviewing the research articles published in the last 10 years, it seems that bilberry leaves have been slightly forgotten, although their phytochemical and pharmacological characteristics are unique.

## 1. Introduction

*Vaccinium myrtillus* is a shrub from the Ericaceae family, genus *Vaccinium*, that grows in mountainous regions in Europe, Asia and North America. The leaves are used in traditional medicine of different countries for the management of diabetes. Until presently, there is no relevant information, only assumptions, regarding the compounds that are responsible for this effect [1]. In the European Pharmacopoeia, as well as in the British Pharmacopoeia, there are three monographs related to bilberry fruits (Bilberry fruit, fresh; bilberry fruit, dried; fresh bilberry fruit dry extract, refined and standardized), but none related to the leaves [2]. The European Medicines Agency also included two monographs for bilberry fruits, but none for bilberry leaves [3]. To our knowledge, bilberry leaves (Myrtilli folium) do not have a monograph in the most known Pharmacopoeias.

Since the discovery and definition of oxidative stress, multiple studies were focused on highlighting natural sources that play a protective role against free radicals [4]. At the top of the list are phenolic compounds, for which remarkable antioxidant capacity has been demonstrated in both in vitro and in vivo studies [5]. Among the sources of polyphenolic compounds, species from the Ericaceae family have been noted [6]. Although crop species are an improved variant, for which certain factors can be controlled so that variations in phytochemical content are minor, wild species will always remain an extraordinary source of therapeutically active compounds, as long as harvesting is controlled, without bringing any damage to the ecosystem.

Another important topic in phytotherapy is the antibacterial effects of natural compounds. Due to multidrug-resistant bacterial pathogens, antibiotic drug research is an absolute necessity.

Well documented in the literature, the influence of pedo-climatic conditions is an important factor that is responsible for the noticeable differences among the chemical components of herbal drugs, and also the accumulation of different metals, having significant effects on the quality of plant products. The objective of this systematic review is to present the recent advances regarding the phytochemistry and pharmacology of *Myrtilli folium*, summarizing the latest developments in this field. As bilberry leaves are included in many supplements for diabetes but not limited to them, a careful evaluation of the knowledge status regarding VM leaves is necessary to provide a starting point for future research.

This review was designed according to Prisma guidelines, and it was registered as a systematic review with the registration number INPLASY2022120029 (10.37766/inplasy2022.12.0029). For this systematic review, the main databases were searched using combinations of the following keywords: “*Vaccinium myrtillus*”, “bilberry”, “leaves”. Only the articles from the last 10 years were selected (2012–2022). The articles that contained data for other *Vaccinium* species were excluded, as well as articles that included analysis of the fruits and red leaves, ecophysiology and stress tolerance.

**Figure 1:**
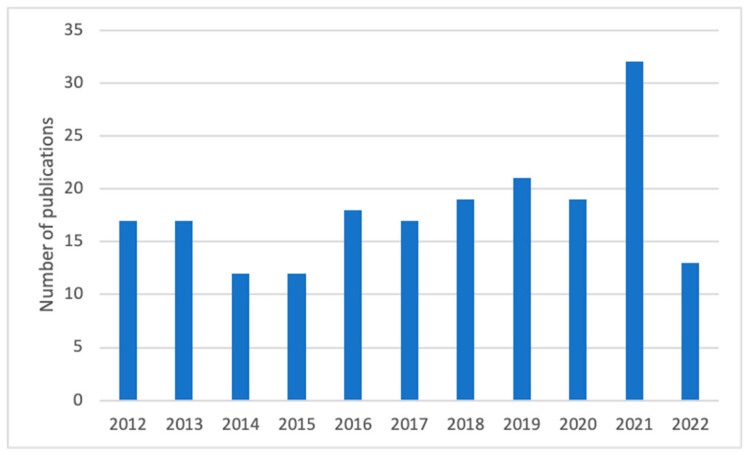
Research tendency containing the terms “*Vaccinium myrtillus*” and “leaves” in publications from 2012 to 31 June 2022 (indexed by the Web of Science Core Collection).

The search performed in the Web of Science Core Collection, PubMed and Scopus (June 2022) led to a total of 197 publications (review, research article, short communications and proceedings paper). All identified articles were pooled to reference manager software (Mendeley) in order to remove duplicates and select relevant papers. After reading the abstract, or in some cases the full text, publications dealing with soil science, ecology, plant pathology and cell culture were excluded, as well as articles about other *Vaccinium* species and *Vaccinium myrtillus* fruits. Following this selection, 64 publications remained, and the distribution over the 10 years is represented in Figure 1. Some of these publications (six research articles) reported the pharmacological action or bioactive compounds of polyherbal formulations/plant mixtures [7,8,9,10], and Ieri et al. reports results for the analysis of the bud extract of bilberry [11].

## 2. Chemical Composition

### 2.1. Total Polyphenols

Bilberry leaves contain a bewildering variety of polyphenolic compounds that are presumed to be responsible for the majority of its therapeutic effects.

The quantitative determination of polyphenolic compounds is a standard determination, which is based on the method developed by Singleton et al. [12]. The results provide valuable information and represent a starting point in a complete phytochemical analysis. The majority of researchers have expressed the contents relative to the dry weight; therefore, the quantitative data obtained in different laboratories can be compared (Table 1). Chemical composition is greatly influenced by the stage of development; the geographical origin, where the country plays a major role not only in the chemical accumulation of secondary metabolites, but also in the pedoclimatic factors, is an important determinant. Not lastly, the reported total polyphenolic content (TPC) is greatly influenced by the method of extraction. As shown in Table 1, the TPC varies within a large range. For the extraction of polyphenols, usually aqueous-alcoholic solvents are used. Optimization methods for extraction of polyphenols have usually demonstrated that 50–70% ethanol gives the best yield.

**Table 1:** Total polyphenolic content reported in several studies.

Concentration mg GAE/g DW	Solvent	Country of Origin	Reference
500–850	80% methanol	Poland	[13]
33–66	40% ethanol	Bulgaria	[14]
76.73	water-glycerin 80:20	Poland	[15]
54.7–106.9	1% aqueous citric acid	Romania	[16]
399.94	acetone-water 70:30	Poland	[17]
173–218	70% ethanol	Montenegro	[18]
35.9–229.8	75% methanol (fresh plant)	Lithuania	[19]
218	70% ethanol	Montenegro	[20]
119.17	water	Bosnia and Herzegovina	[21]
107.79	ethanol		
66.76	ethyl acetate		
99.3	50% ethanol	Ukraine	[22]
68.84	methanol-ascorbic acid-acetic acid	Poland	[23]

GAE—gallic acid equivalents; DW—dry weight.

### 2.2. Total Flavonoids

Flavonoids are an important class of polyphenolic compounds found in large quantities in aerial parts of herbal drugs. Their accumulation is frequently influenced by abiotic and biotic stressors. Compared with TPC, the concentration of flavonoids determined in different regions is less variable as it can be seen in Table 2. Flavonoids are found in plants in two forms: as free aglycones and in glycosidic form. The glycosides have an increased polarity compared with the free aglycones. The forms usually influence the pharmacokinetics of the compound. Of the total flavonoids, quercetin derivatives represent a major percentage, particularly quercetin 3-O-rutinoside and quercetin 3-O-galactoside. Kaempferol glycosides are the second most abundant flavonoids in bilberry leaves. 

### 2.3. Tannin Content

Tannins are an important subgroup of polyphenolic compounds. Herbal products that contain high concentrations of tannins are used in the treatment of nonspecific diarrhea. However, tannins are considered by some authors to be compounds with an antinutrient effect due to the chelating ability of some metals. For example, tannins prevent the absorption of iron in the body, which can lead to iron deficiency anemia in the case of increased consumption or in the case of administration to groups of patients with particular conditions (children, pregnant women, elderly). Quantitative determination of tannins in herbal products is usually performed with an indirect method described in the European Pharmacopoeia, which quantifies total polyphenolic content and the polyphenols not absorbed on standard hide powder [2]. Bilberry leaves contain high concentrations of tannins, usually between 0.8 and 6.7%, as it can be seen in Table 3 [24].

Proanthocyanidins, also known as condensed tannins, are oligomers and polymers of flavan-3-ols. The most encountered monomeric units of proanthocyanidins are (+) catechin and (−) epicatechin, and these units usually have A-type or B-type linkages. B-type proanthocyanidins have either a C4-C8 or a C4-C6 interflavan bond, while A-type proanthocyanidins have a supplementary C2-C7 or C2-C5 ether bond. B-type proanthocyanidins are widely distributed in different herbal drugs, but A-type proanthocyanidins are found only in a few plants, such as *Vaccinium macrocarpon, Cinnamomum sp.,* etc. [25,26,27]. Bilberry leaves contain mainly procyanidins, and the concentration of these compounds in the samples is influenced by the development stage [6]. There are only a few reports in the last 10 years regarding the concentration of proanthocyanidins in the leaves, and the reported concentrations are between 2 and 25.5 mg/g. as shown in Table 4.

### 2.4. Individual Compounds Identified in Vaccinium myrtillus Leaves

VM leaves have a complex and unique composition, and phenolics represent the major constituents. In Table 5 and Table 6, the chemical compounds identified in bilberry leaves are presented. Among polyphenols, hydroxycinnamic acids, flavanols, flavonols, flavonolignans, and even traces of anthocyanins have been identified. Besides phenolic compounds, small amounts of triterpenes and phytosterols can be found in the leaves, and until now, more than 70 individual compounds have been identified. Quantitatively, hydroxybenzoic acids and hydroxycinnamic acid derivatives are dominant in the extracts.

As previously described, there are many differences regarding the concentration of chemical compounds in herbal drugs collected from different areas. Environmental factors are probably responsible for the majority of these differences, observed mostly in the relative proportions of individual compounds, as well as in the presence or absence of some constituents. Extraction conditions also influence the phytochemical profile of the extracts; for example, high temperatures and alkalinity induce epimerization of epicatechin to catechin [28]. 

Chlorogenic acid, also known as 3-O-Caffeoylquinic acid, is the ester of caffeic acid and quinic acid, and along with feruloylquinic acid, it is the dominant phenolic acid in VM leaves [18,29,30]. According to Tian et al., the main phenolic compounds found in the analyzed samples collected from Finland were Caffeoylquinic acids, with 3-O-Caffeoylquinic acid being the dominant compound [31].

Interestingly, gallic acid was reported in significant amounts only in samples from Turkey [32]. Bljajic et al. reported a high concentration of protocatechuic acid in the hydrolyzed extracts [33]. 

**Table 5:** Individual polyphenols identified in bilberry leaves.

**Hydroxycinnamic Acids**	**Concentration Range** **mg/g DW**	**Country of Origin**	**Reference**
Chlorogenic acid	10.04–22.34	Bulgaria	[14]
	3.34–5.94	Romania	[29]
	11.21–20.82	Bosnia and Herzegovina	[33]
	45.51–59.7	Montenegro	[18]
	0.07	Macedonia	[34]
	1.25–4.14	Turkey	[32]
	104.7	Romania	[30]
	n.q.	Finland	[35]
	n.q.	Poland	[36]
	n.q.	Italy	[11]
Neochlorogenic acid	0.34–3.72	Montenegro	[18]
	2.2	Macedonia	[34]
	n.q.	Italy	[11]
Caffeic acid	2.67–5.9	Bulgaria	[14]
	1.25–1.95	Montenegro	[18]
	3.3–28.2	Turkey	[32]
	3.06	Romania	[30]
	n.q.	Poland	[23]
	n.q.	Italy	[11]
p-Coumaric acid	6.37–12.95	Bulgaria	[14]
	0.32–11.9	Bosnia and Herzegovina	[33]
	1.26–2.08	Montenegro	[18]
	8.5–17.6	Turkey	[32]
	n.q.	Italy	[11]
p-Coumaroylquinic acid	0.07	Macedonia	[34]
	n.q.	Italy	[11]
p-Coumaroylhexoside	n.q.	Romania	[16]
Ferulic acid	0.11–0.28	Montenegro	[18]
	3.1–10	Turkey	[32]
*trans*-Ferulic acid	10.95–24.9	Turkey	[32]
Feruloylquinic acid	47.66–59.65	Romania	[29]
3,4-Dicaffeoylquinic acid	0–4.05–5.01	Romania	[29]
3-O-Caffeoylshikimic acid	n.q.	Romania	[16]
	n.q.	Italy	[11]
Sinapic acid	0.18–0.63	Montenegro	[18]
**Hydroxybenzoic acids**	**Concentration range** **mg/g DW**	**Country of origin**	**Reference**
Gallic acid	0.54–0.8	Montenegro	[18]
	67.4–352.3	Turkey	[32]
Syringic acid	32.5–203	Turkey	[32]
Protocatechuic acid	176.45	Bosnia and Herzegovina	[33]
	1.4–1.74	Montenegro	[18]
Vanillic acid	20.8–1156.8	Turkey	[32]
**Other polyphenols**			
Pyrogallol	2.45–3.46	Montenegro	[18]
Resveratrol	4.6–5.15	Montenegro	[18]
	1.5–8.9	Turkey	[32]
**Flavanols**	**Concentration range** **mg/g DW**	**Country of origin**	**Reference**
Gallocatechin	4.84–15.37	Romania	[29]
	n.q.	Hungary	[37]
Epigallocatechin	n.d.–6.56	Romania	[29]
	18.2–197.8	Turkey	[32]
	n.q.	Hungary	[37]
Catechin	4.79–9.87	Romania	[29]
	7.3–95.6	Turkey	[32]
	2.32	Romania	[30]
	n.q.	Finland	[35]
	0.21	Macedonia	[34]
	n.q.	Hungary	[37]
Epicatechin	n.d.–9.66	Romania	[29]
	4.38–5.75	Montenegro	[18]
	10.1–84.1	Turkey	[32]
	1.95	Romania	[30]
	n.q.	Romania	[16]
	n.q.	Finland	[31,35]
	n.q.	Hungary	[37]
Procyanidin B2	0.31–1.03	Montenegro	[18]
Procyanidin dimer I	5.55	Romania	[30]
Procyanidin dimer II	8.7–12.68	Romania	[29]
Procyanidin trimer	10.09–24.30	Romania	[29]
Procyanidin trimer B	n.q.	Italy	[11]
B-type procyanidin dimer I	n.q.	Finland	[31,35]
B-type procyanidin dimer II	n.q.	Finland	[35]
B-type procyanidin dimer III	n.q.	Finland	[35]
Type A/B procyanidin trimer	n.q.	Finland	[35]
	n.q.	Italy	[11]
A-type procyanidin trimer	n.q.	Estonia	[38]
B-type procyanidin trimer	n.q.	Finland	[35]
	n.q.	Estonia	[38]
Proanthocyanidin tetramer B	n.q.	Italy	[11]
Proanthocyanidin pentamer B	n.q.	Italy	[11]
**Flavonols**	**Concentration range** **mg/g DW**	**Country of origin**	**Reference**
Luteolin 5-O-rutinoside	n.q.	Hungary	[37]
Myricetin	49.4–237.6	Turkey	[32]
Quercetin 3-O-rutinoside	42.24–49.83	Romania	[29]
	4.73–4.94	Montenegro	[18]
	0.87	Macedonia	[34]
	n.q.	Hungary	[37]
	n.q.	Italy	[11]
Quercetin 3-O-glucoside	1.29–2.37	Romania	[29]
	9.92–16.2	Montenegro	[18]
	14.5	Romania	[30]
	n.q.	Romania	[16]
	n.q.	Italy	[11]
Quercetin 3-O-rhamnoside	n.q.	Finland	[35]
	1.65	Romania	[30]
	n.q.	Romania	[16]
	0.11	Macedonia	[34]
Quercetin-acetyl-rhamnoside	12.67–18.6	Romania	[29]
	0.98	Romania	[30]
Quercetin 3-O-arabinoside	1.39–1.55	Romania	[29]
	0.64	Romania	[30]
	0.21	Macedonia	[34]
	n.q.	Finland	[35]
	n.q.	Italy	[11]
Quercetin-xyloside	1.3–1.53	Romania	[29]
Quercetin glucosyl-xyloside	0.7	Romania	[30]
Quercetin-diglucoside	0.17–1.42	Romania	[29]
Quercetin 3-O-galactoside	32.16–44.43	Bosnia and Herzegovina	[33]
	2.38–2.55	Montenegro	[18]
	2.45	Macedonia	[34]
	n.q.	Finland	[31,35]
	n.q.	Romania	[16]
	n.q.	Hungary	[37]
	n.q.	Poland	[23]
Quercetin 3-O-glucuronide	n.q.	Finland	[31,35]
	n.q.	Hungary	[37]
	n.q.	Italy	[11]
Quercetin-hexuronide	n.q.	Romania	[16]
Quercetin 3-sambubioside	n.q.	Hungary	[37]
Quercetin	1.16–3.69	Romania	[29]
	24.75–85.64	Bosnia and Herzegovina	[33]
	1.16–7.27	Montenegro	[18]
	2.1–11.4	Turkey	[32]
	n.q.	Hungary	[37]
Kaempferol 3-O-glucoside	1.38–1.6	Montenegro	[18]
Kaempferol 3-O-glucuronide	n.q.	Finland	[35,39]
	n.q.	Italy	[11]
Kaempferol-rhamnoside	n.q.	Finland	[35]
Kaempferol-hexuronide	n.q.	Romania	[16]
Kaempferol	3.45	Bosnia and Herzegovina	[33]
	0.03–0.26	Montenegro	[18]
	1.6–3.4	Turkey	[32]
Taxifolin	n.q.	Hungary	[37]
Flavanolignans			
Cinchonain I	n.q.	Italy	[11]
Cinchonain II	n.q.	Italy	[11]
**Anthocyanins**	**Concentration range** **µg/g DW**	**Country of origin**	**Reference**
Cyanidin-glucoside	n.d.–0.29	Romania	[29]
	n.d.–1.06	Turkey	[32]
Cyanidin-arabinoside	n.d.–0.3	Romania	[29]
Cyanidin-acetyl-glucoside	n.d.–0.33	Romania	[29]
Malvidin 3-O-glucoside	n.d.–1.2	Turkey	[32]

n.d.—not detected; n.q.—compounds were only identified but not quantified.

**Figure 2:**
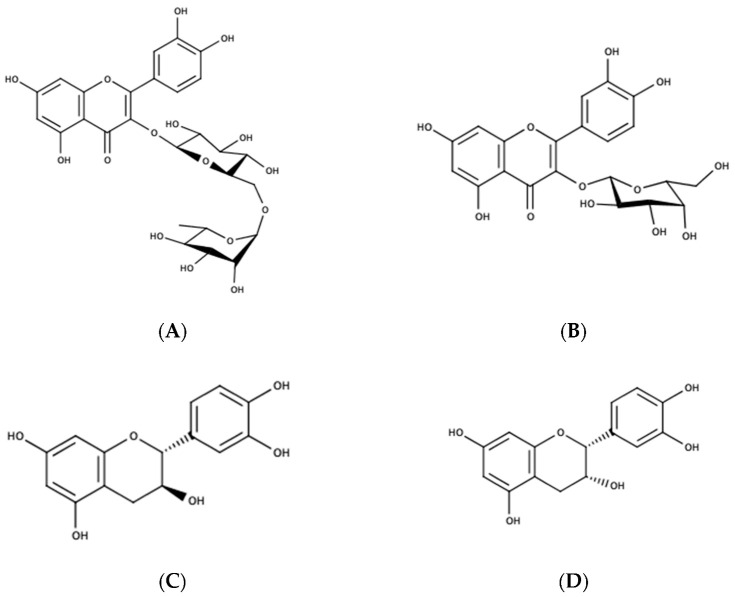

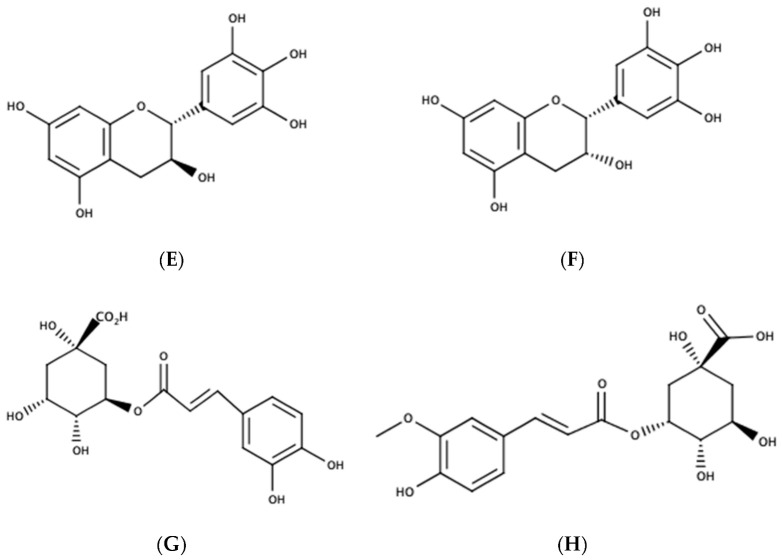
Chemical formulas of the main polyphenols found in bilberry leaves; (**A**)—Quercetin 3-O-rutinoside; (**B**)—Quercetin 3-O-galactoside; (**C**)—Catechin; (**D**)—epicatechin; (**E**)—gallocatechin; (**F**)—epigallocatechin; (**G**)—Chlorogenic acid; (**H**)—Feruloyquinic acid.

Flavonol glycosides found in bilberry leaves have either quercetin or kampferol as aglycones (Figure 2). 

Proanthocyanidins are classified according to the monomeric units in 13 classes, but procyanidins, prodelphinidins and pelargonidins are frequently encountered. Proanthocyanidins with a degree of polymerization between 2–4 are considered oligomers, and those with a degree of polymerization greater than 4 are considered polymers, but this classification is still under debate [40,41]. As shown in Table 5, the proanthocyanidins found in *V. myrtillus* leaves are mainly B-type dimers and trimers procyanidins. These compounds are cleaved through acid hydrolysis into flavan-3-ols. 

Anthocyanins are not a class of compounds that are usually found in bilberry leaves. However, depending on the sun exposure, small quantities of anthocyanins have been detected in bilberry leaves due to a complex regulatory mechanism, influenced by high solar radiation [42]. As well, the presence of anthocyanins could be related to the extraction method, which could have released the anthocyanins from procyanidins.

**Table 6:** Triterpenes and phytosterols identified in bilberry leaves.

Compound	Concentration Range µg/g DW	Country of Origin	Reference
**Triterpenes**			
Oleanolic acid	505–655	Bulgaria	[43]
	853.2	Finland	[44]
	873.6	Poland	[44]
Ursolic acid	377–815	Bulgaria	[43]
	747.7	Finland	[44]
	776.2	Poland	[44]
Lupeol	20–55	Bulgaria	[43]
	24.6	Finland	[44]
	63.4	Poland	[44]
α-Amyrin	102–568	Bulgaria	[43]
	711.9	Finland	[44]
	631.4	Poland	[44]
α-Amyrenone	11.7	Finland	[44]
	22.2	Poland	[44]
β-Amyrin	919.1	Finland	[44]
	987.2	Poland	[44]
β-Amyrenone	15.4	Finland	[44]
	34.7	Poland	[44]
	22.8	Poland	[44]
Hydroxyoleanolic acid	50.1	Finland	[44]
	164.7	Poland	[44]
Hydroxyursolic acid	33.9	Finland	[44]
	115.8	Poland	[44]
**Phytosterols**			
Cycloartanol	20.3	Finland	[44]
Campesterol	11.1	Finland	[44]
	16.7	Poland	[44]
Sitostanol	6.9	Finland	[44]
	8.2	Poland	[44]
Sitosterol	610.9	Finland	[44]
	671.4	Poland	[44]
Stigmastadienone	38.3	Finland	[44]
	20.8	Poland	[44]
Stigmasterol	5.4	Finland	[44]
	5.2	Poland	[44]

Regarding the content of triterpenoids, it seems that most of them are present in free form as neutral triterpenes (β-amyrin and α-amyrin are quantitatively dominant), as triterpenic acids and as steroids [44]. Among triterpenic acids, oleanolic and ursolic acids are found in high concentrations; both are pentacyclic lipophilic compounds that are found ubiquitously in herbal drugs. These acidic triterpenes are found in bilberry leaves extracts only when organic solvents are used [45]. The free terpenoids are accompanied by a small amount of esters (mostly esters of α-and β-amyrin) [44].

Savych et al. reported on the leaves of bilberry saturated fatty acids (palmitic acid in major quantity) and polyunsaturated acids (linoleic and linolenic) [8].

Although the leaves only contain a small amount of essential oil, the volatile compounds identified from the oil are known compounds with potent antibacterial activity. The three predominant terpenes in the essential oil have been identified as 1,8-cineole, alfa-pinene and linalool [46].

### 2.5. Macroelements and Trace Elements

Accumulation of heavy metals in plants due to contamination of the environment can affect the entire food chain. *Vaccinium myrtillus* is one of the plant species that is a good indicator of contaminated soil. Although this capacity is important for environmental research, it has a tremendous negative effect when the herbal products are used as nutraceuticals. The most commonly encountered heavy metals in herbal drugs are usually arsenic, lead and mercury [47]. 

Eeva et al. have evaluated the effects of metal-polluted environments on the accumulation of heavy metals in bilberry leaves. It seems that this species is a good indicator of polluted environments, as the plant tends to accumulate nonessential elements, including toxic elements from the contaminated soil (Table 7). These findings are supported by the results obtained by Kandziora-Ciupa et al. [48], which indicated that in polluted areas, the concentration of Cd and Pb in the leaves severely exceeds the normal limits. *European Pharmacopoeia 10th Edition* limits the concentration of lead in herbal drugs to a maximum of 5 ppm [2]. According to the FDA, the Interim Reference Level (IRL) for lead is 12.5 µg/day for an adult and 3 µg/day for children [49]. In the body, lead is rapidly absorbed and distributed in the central nervous system, organs and bones, and can induce severe lesions at the distribution sites [50]. However, during extraction, only an aliquot of the metal content is extracted, and the yield is dependent on the extraction method. As Brasanac-Vukanovic et al. reported for the extracts obtained from the leaves collected in Montenegro, the lead content in the extracts is below the recommended daily allowance [18]. Overall, special attention should be given when choosing the collection sites, which have to be far from any industrial field or any other source of pollution.

The effects of VM leaves as a complementary treatment for diabetes have been correlated in traditional medicine with the chromium content. Trivalent chromium is considered to be an insulin sensitizer by modulating the phosphorylation of insulin-binding receptors [55,56]. Nevertheless, as shown in Table 7, the concentration of chromium in leaves is low; therefore, the effects cannot be entirely attributed to the chromium content.

## 3. Pharmacokinetics of Polyphenols

**Figure 3:**
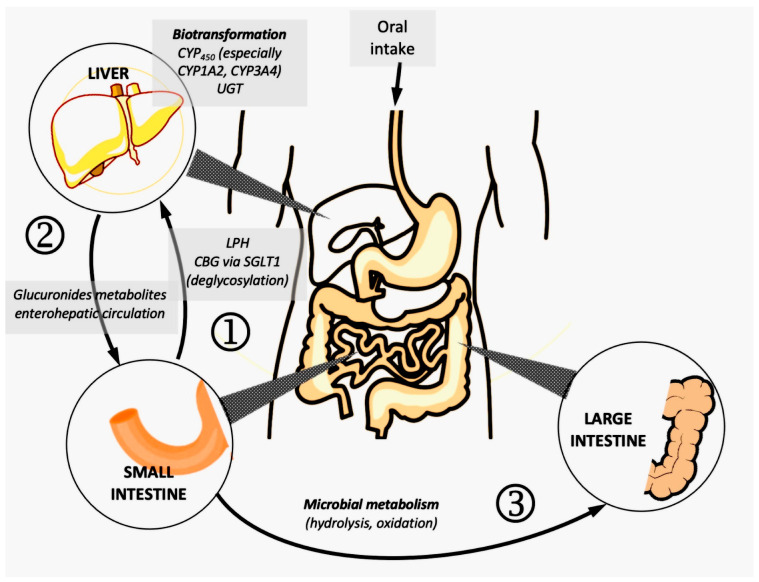
Polyphenols pharmacokinetics; 1—absorption and biotransformation in the intestinal wall; 2—liver biotransformation and enterohepatic circulation through biliary elimination and reabsorption in the duodenum; 3—enzymatic processes under the action of saprophytic flora.

Natural polyphenolic compounds are often found in glycosidic form, and the hydrophilic/lipophilic ratio is strongly modified compared to the parent aglycone; the presence of groups derived from monosaccharides profoundly modifies the pharmacokinetic properties, and the type of glycosidic bond (O-glycoside, C-glycoside) is also important [27]. Broadly speaking, it can be said that natural polyphenolic derivatives (both phenol-carboxylic acids and flavonoids, such as flavonols, flavones, stilbenes, flavanones, etc.) have pharmacokinetic characteristics unfavorable to oral absorption. In reality, however, things are not so simple, and pharmacokinetic studies are difficult to perform, given several specific aspects:

- the importance of the food matrix in the case of the administration of natural extracts, as well as the presence of dimer or trimer forms for catechins and proanthocyanidins (in general, analytical methods only quantify the monomers present in the plasma in bioavailability studies) [57];
- the metabolic processes of deglycosylation that take place at the level of the enterocyte, which lead to the formation of aglycones: intracellular capture by SGLT1 (sodium-glucose co-transporter 1), followed by hydrolysis under the action of CBG (cytosolic beta-glucosidase), respectively, LPH (lactase-phlorizine hydrolase);
- biotransformation in the intestinal wall (via the cytochrome P450 isoform CYP3A4) and in the liver (many CYP450 isoenzymes, but mainly CYP3A4), which gives oxidized metabolites of the initial aglycones; subsequently, the latter can undergo phase II reactions and conjugation with endogenous compounds: glucuronides are obtained (under the action of UGT-uridine-5’-diphosphate glucuronyl transferase), sulfates via SULT (sulfotransferases, with PAPS as co-factor—phospho-adenosyl-phosphosulfate), respectively, compounds methylated via COMT (catechol-O-methyltransferase), as shown in Figure 3 [58];
- glucuronide and sulfate metabolites may undergo enterohepatic circulation due to biliary elimination and reabsorption in the duodenum; some of the compounds already modified can undergo enzymatic hydrolysis and reabsorption in the portal vein;
- the unabsorbed polyphenolic fraction reaches the colon, where it undergoes complex enzymatic processes under the action of saprophytic flora; the newly formed compounds can be absorbed and will be available in the systemic circulation [59].

As a result, despite the reduced bioavailability of natural polyphenolic compounds administered orally, the possibilities of obtaining structurally modified derivatives (deglycosylated, oxidized, methylated, glucuronidated, sulfated) in the small intestine and colon are multiple, and part of them can be subsequently absorbed. Moreover, some pharmacological effects may be indirect by modifying the microbiota of the large intestine and by modulating certain immunopharmacological effects. It is, therefore, difficult to find a simple relationship between the quantity of polyphenols ingested and the pharmacological effects observed, in particular in the case of long-term treatments (where the total cumulative dose depends on the factors mentioned above) [5,58,59,60].

Ștefănescu et al. have investigated the bioaccessibility of phenolic compounds from bilberry leaves using simulated in vitro digestion. The research aimed to compare the bioaccessibility of the extract to the microencapsulation. The results indicated that the microencapsulated spray dried extract had a better response compared with the solution. The results also indicated that the saliva phase had a minimal effect on the phenolic profile, while the gastric and intestinal phases lead to a significant decrease of the analyzed phenolic compounds [30]. 

## 4. Pharmacological Activity

According to scientific literature, bilberry leaves are endowed with multiple therapeutic effects, such as anti-diabetic, hypolipidemic, antibacterial, antiviral, anticarcinogenic and anti-inflammatory. In vitro and preclinical investigations provide the majority of the evidence; to our knowledge, no clinical studies have been carried out in the last 10 years.

The activity of the extract is correlated with the phytochemical profile, and a structure-activity relationship can be observed. This relationship mainly influences the antioxidant activity, but subsequently will influence the other related effects. It has been observed that for polyphenol carboxylic acids, the antioxidant activity decreases in the order hydroxy phenylacetic acid > hydroxycinnamic acid > hydroxybenzoic acid, because the electron donating ability decreases in the same trend. Additionally, the number of hydroxy and methoxy groups is proportional to the antioxidant effects. Considering the large class of flavonoids, the presence of the C2=C3 double bond and the OH-linked in the C3 position is essential for the antioxidant activity. Moreover, the C2=C3 double bond seems to be crucial for other effects, such as aldose-reductase inhibition, alpha-amylase and alpha-glucosidase inhibition. The glycosylation of flavonoids plays an important role in the structure-activity relationship, but sometimes the effects of flavonoids can be unpredictable because of their individual pharmacokinetic profile. Usually, the aglycone is more active than the corresponding glycosides, an effect noticed by correlating the in vitro data with the in vivo data: glycosides produced an effect only in vivo, while aglycones produced the effect both in vitro and in vivo. Considering these observed effects, it can be concluded that metabolization of the glycosides highly influences the pharmacological activity [61,62].

### 4.1. Antioxidant Capacity

Antioxidant activity is the capacity of a compound to neutralize reactive oxygen species (ROS) and reactive nitrogen species (RNS), such as superoxide anion radical (O_2_^•−^), hydroxyl radical (HO•), hydrogen peroxide (H_2_O_2_), nitric oxide (•NO), nitrogen dioxide (NO_2_^−^) and peroxynitrite (OONO^−^). These extremely reactive molecules can occur during normal cellular activity, and in normal conditions, these are neutralized by the organism’s antioxidant systems, such as superoxide dismutase (SOD), glutathione peroxidase (GPx) and catalase (CAT). Often, an imbalance occurs between the generation of reactive oxygen systems and the antioxidant system, leading to oxidative stress (OS) and, further, to a higher oxidation rate of lipids and proteins with severe consequences at the cellular level [63]. 

According to the literature, phenolic compounds can scavenge free radicals through four different chemical pathways: proton coupled electron transfer, electron transfer–proton transfer, sequential proton loss electron transfer and adduct formation. The reaction environment determines how these processes interact in equilibrium [64].

Because of the numerous polyphenolic compounds found in bilberry leaves, the extracts have high antioxidant activity. Tian et al. have evaluated the antioxidant effects of bilberry leaves and compared the results with other extracts form berries, leaves and branches of berry plants. Their results indicated that the leaf extracts have higher antioxidant activity compared with the berry extracts from the same species, and also the bilberry extracts were among the most powerful antioxidants from the tested leaf extracts [65]. Teleszko et al. conducted a similar study, and their results showed that bilberry leaf extracts had moderate antioxidant effects compared with other extracts [23].

### 4.2. Antimicrobial Activity

Natural compounds are considered promising agents with antibacterial activity. Numerous mechanisms of action have been proposed, such as the inhibition of bacterial binding to cell walls (antiadhesion effect), disintegration of the liposaccharide layers (outer membrane damage), destruction of the cytoplasmatic phospholipid bilayer (cell wall damage) and complexation of metal ions [17]. Besides their direct action, natural compounds can act in synergy with allopathic antibiotic medication, an interaction demonstrated through the reduction of the minimum inhibitory concentration of the antibiotic when combined with natural compounds or extracts. The synergistic effects reside in the ability of some natural compounds (e.g., polyphenols) to increase bacterial membrane permeability, thus facilitating the cellular entrance of antibiotics [17,66]. Moreover, this combinational therapy can decrease the development of antibacterial resistance [66]. 

Gram-negative bacterial strains are usually more resistant compared to gram-positive bacteria due to the conformation of their membrane, slowing the antibiotic in reaching the site of action [67].

Tian et al. evaluated the antibacterial effects of VM extracts on *Staphylococcus aureus*, *Bacillus cereus*, *Listeria monocytogenes*, *Escherichia coli* and *Salmonella enterica* sv. *Typhimurium*. No inhibitory activity was observed in Staphylococcus aureus and *E. coli*, and also weak efficacy against *Bacillus cereus* was recorded. However, moderate efficacy was noticed against *Salmonella* *enterica* sv. *Typhimurium* [65]. The authors also demonstrated synergistic activities with linezolid, an oxazolidinone derivative that inhibits protein synthesis by binding to a site of the 50S ribosomal subunit. A weaker synergistic effect was recorded with vancomycin, a tricyclic glycopeptide that inhibits cell wall synthesis. Ștefănescu et al. also evaluated the effects of VM extracts on different bacterial strains (Table 8). In opposition to the results obtained by Tian et al., their results indicated a good inhibitory effect on *S. aureus*. Analyzing the methodology of both research papers, it can be seen that the chemical profile of the extracts is completely different, and also different strains were used. Therefore, the results can be explained by the different research methodology. In one of the research projects, the extracts were obtained from dried herbal drugs, while in the other, the extracts were obtained from fresh drugs. Acidified aqueous ethanol was used for the fresh drug (ethanol:water:acetic acid, 70:30:1, *v*/*v*/*v*), while 40% ethanol was used for the dried drug. For Rhodococcus equi, the antibacterial activity of VM extracts was similar to that of the bactericidal aminoglycoside Streptomycin.

Louis Rice has abbreviated the most problematic pathogens “ESKAPE”, after their names: *Enterococcus faecium, Staphylococcus aureus, Klebsiella pneumoniae, Acinetobacter baumanni, Pseudomonas aeruginosa* and *Enterobacter* species [69]. These bacterial strains are responsible for the most nosocomial infections, and their resistance to different antibiotics is quite concerning. As shown in Table 8, different research has demonstrated the antibacterial activity of *Vaccinium myrtillus* leaf extract on four out of the six “ESKAPE” pathogens. However, these findings were demonstrated in vitro; therefore, the research should be extended in preclinical and clinical studies in order to obtain valid research.

Aflatoxins are mycotoxins, metabolic products of *Aspergillus flavus* and *A. parasiticus*. Acute exposure to these aflatoxins results in nausea, vomiting, abdominal pain and sometimes convulsions. Chronic exposure usually results in hepatotoxicity and immunotoxicity [70].

Vamvakas et al. have evaluated the effects of methanolic and aqueous extracts on the synthesis of aflatoxin by *Aspergillus flavus*. Their results indicate that the aqueous extracts totally inhibit aflatoxins synthesis, suggesting the potential role of bilberry leaf extracts as food additives [71].

### 4.3. Metabolic Modulation

Diabetes is a metabolic disorder characterized by an imbalance in glucose metabolism, as well as in lipid and protein metabolism. Type 2 diabetes is associated with glucose resistance and a relative secretory deficiency of β-pancreatic cells. The prevalence of type 2 diabetes is increasing at an alarming rate. Uncontrolled hyperglycemia leads to the activation of several pathways, such as the polyol and hexosamine pathway, further leading to the formation of advanced glycation end-products, activation of protein kinase C and accumulation of reactive oxygen species, causing diabetes-specific complications, such as retinopathy, nephropathy and neuropathy [72].

The major goal of therapy is to maintain glycemic levels within normal limits and to prevent long-term complications. At the onset of the disease, a lifestyle intervention is needed because it could significantly influence the progression of the disease [73]. 

Although pharmacological therapy remains the main treatment, herbal drugs play an important role in the management of diabetes because therapy supplementation with natural compounds results in better glycemic control and improvement of diabetes-specific symptoms and complications. The desirable situation is when herbal therapy is recommended at the onset of the disease and combined with lifestyle intervention. 

As bilberry leaves have been used for centuries in traditional medicine for diabetes and are classified in phytotherapy books as antidiabetic herbal drugs, various research was conducted in order to evaluate the effects of the extracts and to describe the possible mechanisms of action, but recent research regarding these effects is scarce.

Alpha-amylase is an enzyme secreted by the salivary glands and pancreas that hydrolyzes polysaccharides into shorter oligosaccharides in the gastrointestinal tract. Alpha-glucosidase is a carbohydrate-hydrolase that hydrolyzes disaccharides to glucose, which is responsible for elevated post-prandial hyperglycemia. There are three synthetic α-glucosidase inhibitors approved for diabetes treatment: acarbose, voglibose and miglitol. However, due to their side effects (bloating, diarrhea, flatulence, gastrointestinal discomfort), they are rarely used in diabetes treatment [74]. These gastrointestinal side effects are caused by the fermentation of undigested carbohydrates. An impressive number of reports showed that natural compounds have strong inhibition activity on these enzymes. Different flavonoids, tannins, catechins and polyphenol carboxylic acids can actively inhibit α-amylase and α-glucosidase [74,75,76]. Interestingly, the gastrointestinal side effects of herbal extracts used as enzyme inhibitors are less frequent, and we speculate that the existing compounds in the total extract can suppress the occurrence of these side effects. Tannins, for example, have been used for centuries in the treatment of unspecific diarrhea due to their astringent effect [77]. This effect combined with the potent α-amylase and α-glucosidase activity could lead to a better outcome.

Bljajic et al. reported that the tested extracts (aqueous and hydroethanolic) from VM leaves had an excellent α-glucosidase inhibitory capacity, and the highest inhibition was recorded for the hydroethanolic extract. The same authors reported that the tested extracts did not show any observable effect on α-amylase [33]. However, Takacs et al. reported similar results for both α-amylase and α-glucosidase inhibition assays. Flavan-3-ols are also reported to strongly inhibit α-amylase and α-glucosidase [78].

In a rat animal model, the efficacy of the aqueous extract of VM leaves in reducing a rise in blood glucose following starch consumption was observed in both obese rats and those with prediabetes or streptozocin-induced diabetes, the efficacy of the extract being comparable to that of acarbose [37].

Human hepatocarcinoma cell line (Hep G2) is frequently used in biochemical studies because it has common characteristics with human hepatocyte in culture. Bljajic et al. investigated the effect of bilberry leaf extracts on glucose-induced oxidative stress in Hep G2 cells, and the results showed that the aqueous extracts are capable of restoring the GSH levels [33]. We have previously discussed in Section 4.1 the antioxidant effects of VM leaf extracts.

Sidorova et al. compared the effect of bilberry leaves and *Phaseolus vulgaris* seed extracts on diabetic rats. However, because the extracts were introduced in the drinking water, the dosing methodology is ambiguous [79]. 

In our previous study, we demonstrated that *Vaccinium myrtillus* leaves are capable of reducing the glucose level of diabetic rats [80]. In addition, the oral treatment with VM leaf extracts increased insulin secretion after eight weeks of treatment. We presumed that the extracts have a secretagogue effects and/ or could restore beta-cells function. The four flavonoids found in larger quantities in VM leaves—myricetin, quercetin 3-O-rutinoside, quercetin 3-O-galactoside and quercetin—all modulate insulin secretion [81,82,83]. Moreover, chlorogenic acid stimulates glucose uptake in skeletal muscle by activating AMP-kinase [84], and other phenolic acids also stimulate glucose uptake [73].

Several studies have investigated the antihyperglycemic potential of multi-herbal formulas that contained bilberry leaves, among other herbs [9,17,37]. The limitations of these studies are that the possible effects observed are difficult to interpret because of the numerous compounds, and the unpredictable interactions between the compounds in the formulation, but also during absorption, distribution and metabolization.

Additionally, the antihyperglycemic effects of VM leaf extracts can prevent cataract development in diabetic rats, as we previously proved in one of our research projects. Considering the inhibition activity of chlorogenic acid on aldose reductase, the enzyme that catalyzes on the polyol pathway, we can presume that the transformation of glucose into sorbitol, a polyol with osmotic properties, is a mechanism of action that explains the anticataractogenic effect [80,85].

Accordingly, bilberry leaf extracts can favorably influence the lipid profile and can delay the onset of other diabetic complications, such as liver impairment and nephropathy [86].

Zagayko et al. evaluated the effect of 3 weeks treatment with bilberry leaf extracts on obese hamsters. The study concluded that in the group treated with bilberry leaves, a reduction of weight was observed, and also the visceral fat mass and serum lipid profile were reduced in a dose-dependent manner [22].

### 4.4. Dermatological Effects

Incorporation of natural phenolics in cosmeceutical formulations concerns many researchers in the cosmetics industry. Consumers’ expectations of natural ingredients as active ingredients in cosmetics is increasing, along with the unsaturated need for innovation in this area. 

Tadic et al. evaluated the effects of a cream with 6% VM extract and 6% bilberry seed oil on the skin of healthy volunteers, and the results indicated that the cream with bilberry leaf extract significantly improved the hydration of the skin compared with the group that was treated only with the base without the active ingredients [20].

The results of in vitro and in vivo studies using bilberry leaf extracts are quite encouraging, as shown in this section, but are not yet supported by clinical studies. Although pharmacokinetic studies for individual compounds are widely available in scientific literature, pharmacokinetic studies for extracts are crucial for determining the therapeutic dose of the extract. Regarding the use of VM leaf extracts in diabetes treatment, pharmacokinetic studies should also be carried out in patients with diabetes, as it has been proved that the pharmacokinetics and pharmacodynamics of drugs is modified in subjects with diabetes. The absorption of drugs in diabetic subjects is affected by the modified gastric emptying time, which is caused by the reduced gastric mucosal blood flow due to the micro/macrovascular complications and autonomic neuropathy or by the gastroparesis [87]. The distribution is modified due to the altered protein binding and the shift in the volume of distribution. The metabolism is influenced by the differential expression of a variety of CYPs [88]. The elimination rate is also modified in chronic kidney diseases [89]. Additionally, the evaluation of herb-drug interactions involving pharmacokinetic and pharmacodynamic interactions are still unknown. All these unanswered questions offer different opportunities for future research.

## 5. Safety Evaluation

Bilberry leaves do not contain toxic compounds per se, but due to the tannin content, precautions must be taken, as tannins can produce constipation. Because of the tannin capacity for forming complexes with proteins and proteases (glycoproteins, gut bacterial enzymes), tannins can prevent the absorption of nutrients and also decrease their digestibility. A very important interaction is caused by long-term treatments with tannins, which can lead to iron deficiency, i.e., tannins preventing the absorption of iron. These precautions are very important, mainly for children and the elderly [1,90].

## 6. Previous Published Reviews

Although the health benefits of anthocyanins from the fruits have been the topic of many reviews, a scanty number of reviews focusing on bilberry leaves were found in the recent published literature.

The review elaborated by Chehri et al. is focused on the effects of both fruits and leaves from bilberry [91], while the review published by Tundis et al. is focused on the chemical composition and biological effects of different *Vaccinium* species [92].

Compared with these reviews, the present review highlighted the latest advances related to *Vaccinium myrtillus* leaf composition and effects. To date, this is the only review published in the last decade that is focused only on bilberry leaves.

Bilberry leaves are easily found in large quantities in the wild flora of many countries. Despite the appealing benefits that these extracts can offer, they are underused and found under the shade of some newly discovered herbal drugs. Considering the importance of evidence-based medicine, clinical studies are mandatory to close the research cycle in order to evaluate the pharmacokinetic and pharmacodynamic profile. Then, standardized extracts in therapeutically active compounds could be used in order to obtain a reproducible therapeutic efficacy and safety in administration.

## 7. Conclusions

The present review merged the information from the recent available scientific reports regarding the phytochemistry and pharmacological effects of *Vaccinium myrtillus* leaves. Surprisingly, a low number of articles were published in the last 10 years regarding the composition and pharmacological effects of *Vaccinium myrtillus* leaves because the majority of previous studies was mostly focused on the fruits.

This article highlights the fact that researchers’ interest in bilberry leaves has declined considerably over the last decade, leaving many questions unanswered.

## Figures and Tables

**Table 2:** Total flavonoid content reported in different studies.

Concentration mg QE/g DW	Country of Origin	Reference
12–35	Bulgaria	[14]
16.36	Poland	[15]
25.92	Poland	[17]
14.6–23.8	Montenegro	[18]
8–40	Lithuania	[19]
21.5	Montenegro	[20]
19.3	Ukraine	[22]

QE—quercetin equivalents.

**Table 3:** Total tannin content reported in different studies.

Concentration % (*w/w*)	Country of Origin	Reference
1.54 (hydrolysable, HPLC)	Poland	[17]
6.49–9.17	Montenegro	[18]
9.17	Montenegro	[20]

**Table 4:** Total proanthocyanidin concentration determined in different VM leaves samples.

Concentration mg/g	Country of Origin	Reference
2–5	Bulgaria	[14]
8.03	Poland	[15]
25.45	Poland	[17]
24.75	Poland	[23]

**Table 7:** Element concentrations in VM leaves collected from different locations.

**Element**	**Concentration**	**Location**	**Reference**
Al	7.45–186.2 µg/g extr	Montenegro	[18]
	0.09 mg/g	Norway	[51]
	98–175 mg/kg	Poland	[13]
As	0.059–0.297 µg/g extr	Montenegro	[18]
	1.02 µg/g	Finland (polluted area)	[52]
	0.02 µg/g	Norway	[51]
B	31 µg/g	Norway	[51]
Ba	5.36–69.6 µg/g extr	Montenegro	[18]
	0.028 mg/g	Norway	[51]
Cd	0.016–0.112 µg/g extr	Montenegro	[18]
	0.09–6.26 µg/g	Poland	[53]
	0.12 µg/g	Finland (polluted area)	[52]
	0.02 µg/g	Norway	[51]
	0.34–9.81 mg/kg	Poland	[13]
	0.81–4.43 mg/g	Poland	[48]
Co	0.055 µg/g extr	Montenegro	[18]
	0.3 µg/g	Finland (polluted area)	[52]
	0.02 µg/g	Norway	[51]
Ni	2.18–4.4 µg/g extr	Montenegro	[18]
	12.9 µg/g	Finland (polluted area)	[52]
	0.4 µg/g	Norway	[51]
	1.93–2.17 mg/g	Poland	[48]
Pb	0.47–0.63 µg/g extract	Montenegro	[18]
	0.0036–0.157 mg/g	Poland	[53]
	0.00085 mg/g	Finland (polluted area)	[52]
	0.0003 mg/g	Norway	[51]
	3.26–106 mg/g	Poland	[48]
	4.55–232.1 mg/kg	Poland	[13]
Sr	6–14.7 µg/g extr	Montenegro	[18]
	0.007 mg/g	Norway	[51]
**Micronutrient**	**Concentration**	**Location**	**Reference**
Cr	0.123–1.11 µg/g extr	Montenegro	[18]
Cu	2.99–33.31 µg/g extr	Montenegro	[18]
	20.6 µg/g	Finland (polluted area)	[52]
	7.3 µg/g	Norway	[51]
	6.29–8.32 mg/g	Poland	[48]
	2.17–9.07 mg/kg	Poland	[13]
Fe	17.4–25.8 µg/g	Montenegro	[18]
	0.045 mg/g	Norway	[51]
	59.1–379.4 mg/g	Poland	[48]
	9.36–410 mg/g	Poland	[53]
	59.6–108.6 mg/kg	Poland	[13]
Mn	251.4–1210 µg/g extr	Montenegro	[18]
	38.8–1139 mg/g	Poland	[53]
	39.9–1124.8 mg/kg	Poland	[13]
	414 µg/g	Finland (polluted area)	[52]
	0.98 mg/g	Norway	[51]
	59.3–416.9 mg/g	Poland	[48]
Mo	0.26 µg/g	Finland (polluted area)	[52]
	0.05 µg/g	Norway	[51]
Se	0.19 µg/g	Finland (polluted area)	[52]
	0.1 µg/g	Norway	[51]
Zn	20.5–31.5 µg/g	Montenegro	[18]
	11.2–207 mg/g	Poland	[53]
	15.6 µg/g	Finland (polluted area)	[52]
	21.7–99.1 mg/g	Poland	[48]
	0.021 mg/g	Norway	[51]
	12.2–332.3 mg/kg	Poland	[13]
**Macronutrient**	**Concentration** **mg/g**	**Location**	**Reference**
K	8.2–17.2	Montenegro	[18]
	13.3	Serbia	[54]
	7.3	Norway	[51]
	3.3–4.99	Poland	[13]
Na	0.138–1.262	Montenegro	[18]
	0.09	Norway	[51]
	1.7–2.2	Poland	[13]
Mg	1.5–3.8	Montenegro	[18]
	0.41	Serbia	[54]
	1.4	Norway	[51]
	0.5–1.3	Poland	[13]
P	1.1	Norway	[51]
	3.13–3.3	Poland	[13]
Ca	1.2–5.9	Montenegro	[18]
	0.007	Finland (polluted area)	[52]
	2.36	Serbia	[54]
	5.6	Norway	[51]
S	1.7	Norway	[51]
	2.94–4	Poland	[13]

**Table 8:** Antibacterial and antifungal activity of VM leaf extracts.

Strain	MIC mg/mL	Reference
**Gram positive**		
*Bacillus cereus*	+	[65]
*Staphylococcus aureus* ATCC 49444	0.06–0.12	[29]
ATCC 29213	1.5	[17]
clinical strain	0.75	
	+	[65]
ATCC 25923	+	[68]
wound swabs	63	[54]
ATCC 6538	63	
*Staphylococcus* *epidermidis*		[54]
wound swabs	15.75	
ATCC 12228	15.75	
*Streptococcus pyogenes*		
ATCC 19615	31.5	[54]
wound swabs	31.5	
*Enterococcus faecalis* ATCC 29212	0.24–0.48	[29]
clinical isolate	20–40	[21]
*Propionibacterium acnae*		
ATCC 11827	126	[54]
*R. equi.* ATCC 6939	0.12	[29]
*Listeria monocytogenes*	+	[65]
**Gram negative**		
*E. coli* ATCC 25922	0.48–0.96	[29]
	+	[68]
clinical isolate	40	[21]
	+	[65]
*K. pneumoniae* DSMZ 2026	0.24–0.48	[29]
ATCC 10031	126	[54]
wound swabs	126	
ATCC 700603	+	[68]
*P. aeruginosa* ATCC 27853	0.48	[29]
	+	[68]
ATCC 9027	31.5	[54]
wound swabs	31.5	
*Salmonella enterica*	+	[65]
*Acinetobacter boumanii*		
ATCC 196060	63	[54]
*Acinetobacter sp.*		
wound swabs	252	[54]
*Proteus mirabilis*		
*ATCC 12453*	31.5	[54]
*wound swabs*	63	
*Proteus vulgaris* clinical isolate	20	[21]
*ATCC 6380*	+	[68]
**Fungi**		
*Candida albicans* *ATCC 10231*	250	[29]
*Candida zeylanoides* *ATCC 20367*	62.5–125	[29]
*Candida parapsilosis* *ATCC 22019*	62.5	[29]

+ positive activity, but no determined MIC.

## Data Availability

Not applicable.

## Abbreviations

VM—*Vaccinium myrtillus*, GAE—gallic acid equivalents, TPC—total polyphenolic content, DW—dry weight, QE—quercetin equivalents, FDA—Food and Drug Administration, IRL—Interim reference level, SGLT1—sodium-glucose co-transporter 1, CBG—cytosolic beta-glucosidase, LPH—lactase-phlorizin hydrolase, SULT—sulfotransferases, COMT—catechol-O-methyltransferase, ROS—reactive oxygen species, OS—oxidative stress, GSH—glutathione, RNS—reactive nitrogen species, MIC—minimum inhibitory concentration.

## References

[1] Evans W. (2009). Trease and Evans’ Pharmacognosy.

[2] Council of Europe (2019). European Pharmacopoeia.

[3] EMA Herbal Medicinal Products. https://www.ema.europa.eu/en/human-regulatory/herbal-medicinal-products.

[4] Ősz B.-E., Jîtcă G., Ștefănescu R.-E., Pușcaș A., Tero-Vescan A., Vari C.-E. (2022). Caffeine and Its Antioxidant Properties—It Is All about Dose and Source. Int. J. Mol. Sci..

[5] Di Lorenzo C., Colombo F., Biella S., Stockley C., Restani P. (2021). Polyphenols and Human Health: The Role of Bioavailability. Nutrients.

[6] Ștefănescu B.E., Szabo K., Mocan A., Crişan G. (2019). Phenolic Compounds from Five Ericaceae Species Leaves and Their Related Bioavailability and Health Benefits. Molecules.

[7] Savych A., Marchyshyn S., Mosula L., Bilyk O., Humeniuk I., Davidenko A. (2022). Analysis of Amino Acids Content in the Plant Components of the Antidiabetic Herbal Mixture by GC-MS. Pharmacia.

[8] Savych A., Basaraba R., Muzyka N., Ilashchuk P. (2021). Analysis of Fatty Acid Composition Content in the Plant Components of Antidiabetic Herbal Mixture by GC-MS. Pharmacia.

[9] Madić V., Petrović A., Jušković M., Jugović D., Djordjević L., Stojanović G., Vasiljević P. (2021). Polyherbal Mixture Ameliorates Hyperglycemia, Hyperlipidemia and Histopathological Changes of Pancreas, Kidney and Liver in a Rat Model of Type 1 Diabetes. J. Ethnopharmacol..

[10] Madić V., Stojanović-Radić Z., Jušković M., Jugović D., Žabar Popović A., Vasiljević P. (2019). Genotoxic and Antigenotoxic Potential of Herbal Mixture and Five Medicinal Plants Used in Ethnopharmacology. S. Afr. J. Bot..

[11] Ieri F., Martini S., Innocenti M., Mulinacci N. (2013). Phenolic Distribution in Liquid Preparations of *Vaccinium myrtillus* L. and *Vaccinium vitis idaea* L.: Analysis of Liquid Preparation of Bilberry and Lingonberry. Phytochem. Anal..

[12] Singleton V.L., Orthofer R., Lamuela-Raventós R.M. (1999). Analysis of Total Phenols and Other Oxidation Substrates and Antioxidants by Means of Folin-Ciocalteu Reagent. Methods in Enzymology.

[13] Kandziora-Ciupa M., Dabioch M., Nadgórska-Socha A. (2021). Evaluating the Accumulation of Antioxidant and Macro- and Trace Elements in *Vaccinium myrtillus* L. Biol. Trace Elem. Res..

[14] Vrancheva R., Ivanov I., Badjakov I., Dincheva I., Georgiev V., Pavlov A. (2021). Intrapopulation Variation of Polyphenolic Compounds with Antioxidant Potential in Bulgarian Bilberry (*Vaccinium myrtillus* L.). C. R. Acad. Bulg. Sci..

[15] Ziemlewska A., Zagórska-Dziok M., Nizioł-Łukaszewska Z. (2021). Assessment of Cytotoxicity and Antioxidant Properties of Berry Leaves as By-Products with Potential Application in Cosmetic and Pharmaceutical Products. Sci. Rep..

[16] Bujor O.-C., Le Bourvellec C., Volf I., Popa V.I., Dufour C. (2016). Seasonal Variations of the Phenolic Constituents in Bilberry (*Vaccinium myrtillus* L.) Leaves, Stems and Fruits, and Their Antioxidant Activity. Food Chem..

[17] Sadowska B., Paszkiewicz M., Podsędek A., Redzynia M., Różalska B. (2014). *Vaccinium myrtillus* Leaves and Frangula Alnus Bark Derived Extracts as Potential Antistaphylococcal Agents. Acta Biochim. Pol..

[18] Brasanac-Vukanovic S., Mutic J., Stankovic D., Arsic I., Blagojevic N., Vukasinovic-Pesic V., Tadic V. (2018). Wild Bilberry (*Vaccinium myrtillus* L., Ericaceae) from Montenegro as a Source of Antioxidants for Use in the Production of Nutraceuticals. Molecules.

[19] Kaškonienė V., Bimbiraitė-Survilienė K., Kaškonas P., Tiso N., Česonienė L., Daubaras R., Maruška A.S. (2020). Changes in the Biochemical Compounds of *Vaccinium myrtillus*, *Vaccinium vitis-idaea*, and Forest Litter Collected from Various Forest Types. Turk. J. Agric. For..

[20] Tadić V.M., Nešić I., Martinović M., Rój E., Brašanac-Vukanović S., Maksimović S., Žugić A. (2021). Old Plant, New Possibilities: Wild Bilberry (*Vaccinium myrtillus* L., Ericaceae) in Topical Skin Preparation. Antioxidants.

[21] Dragana M.V., Miroslav R.P., Branka B.R.G., Olgica D.S., Sava M.V., Ljiljana R.Č. (2013). Antibacterial and Antioxidant Activities of Bilberry (*Vaccinium myrtillus* L.) In Vitro. Afr. J. Microbiol. Res..

[22] Zagayko A.L., Kolisnyk T.Y., Chumak O.I., Ruban O.A., Koshovyi O.M. (2018). Evaluation of Anti-Obesity and Lipid-Lowering Properties of *Vaccinium myrtillus* Leaves Powder Extract in a Hamster Model. J. Basic Clin. Physiol. Pharmacol..

[23] Teleszko M., Wojdyło A. (2015). Comparison of Phenolic Compounds and Antioxidant Potential between Selected Edible Fruits and Their Leaves. J. Funct. Foods.

[24] Hăncianu M., Gîrd C.E., Stănescu U. (2020). Farmacognozie. Produse Vegetale cu Substanțe Bioactive.

[25] Tao W., Zhang Y., Shen X., Cao Y., Shi J., Ye X., Chen S. (2019). Rethinking the Mechanism of the Health Benefits of Proanthocyanidins: Absorption, Metabolism, and Interaction with Gut Microbiota. Compr. Rev. Food Sci. Food Saf..

[26] Rauf A., Imran M., Abu-Izneid T., Iahtisham-Ul-Haq, Patel S., Pan X., Naz S., Sanches Silva A., Saeed F., Rasul Suleria H.A. (2019). Proanthocyanidins: A Comprehensive Review. Biomed. Pharmacother..

[27] Luca S.V., Macovei I., Bujor A., Miron A., Skalicka-Woźniak K., Aprotosoaie A.C., Trifan A. (2020). Bioactivity of Dietary Polyphenols: The Role of Metabolites. Crit. Rev. Food Sci. Nutr..

[28] Valiñas M.A., Lanteri M.L., ten Have A., Andreu A.B. (2017). Chlorogenic Acid, Anthocyanin and Flavan-3-Ol Biosynthesis in Flesh and Skin of Andean Potato Tubers (*Solanum tuberosum* Subsp. Andigena). Food Chem..

[29] Ștefănescu B.-E., Călinoiu L.F., Ranga F., Fetea F., Mocan A., Vodnar D.C., Crișan G. (2020). Chemical Composition and Biological Activities of the Nord-West Romanian Wild Bilberry (*Vaccinium myrtillus* L.) and Lingonberry (*Vaccinium vitis-idaea* L.) Leaves. Antioxidants.

[30] Ștefănescu B.E., Nemes S.-A., Teleky B.-E., Călinoiu L.F., Mitrea L., Martău G.A., Szabo K., Mihai M., Vodnar D.C., Crișan G. (2022). Microencapsulation and Bioaccessibility of Phenolic Compounds of Vaccinium Leaf Extracts. Antioxidants.

[31] Tian Y., Liimatainen J., Alanne A.-L., Lindstedt A., Liu P., Sinkkonen J., Kallio H., Yang B. (2017). Phenolic Compounds Extracted by Acidic Aqueous Ethanol from Berries and Leaves of Different Berry Plants. Food Chem..

[32] Değirmencioğlu N., Gürbüz O., Karatepe G.E., Irkin R. (2017). Influence of Hot Air Drying on Phenolic Compounds and Antioxidant Capacity of Blueberry (*Vaccinium myrtillus*) Fruit and Leaf. J. Appl. Bot. Food Qual..

[33] Bljajić K., Petlevski R., Vujić L., Čačić A., Šoštarić N., Jablan J., Saraiva de Carvalho I., Zovko Končić M. (2017). Chemical Composition, Antioxidant and α-Glucosidase-Inhibiting Activities of the Aqueous and Hydroethanolic Extracts of *Vaccinium myrtillus* Leaves. Molecules.

[34] Stefkov G., Hristovski S., Petreska Stanoeva J., Stefova M., Melovski L., Kulevanova S. (2014). Resource Assessment and Economic Potential of Bilberries (*Vaccinium myrtillus* and *Vaccinium uliginosum*) on Osogovo Mtn., R. Macedonia. Ind. Crops Prod..

[35] Liu P., Lindstedt A., Markkinen N., Sinkkonen J., Suomela J.-P., Yang B. (2014). Characterization of Metabolite Profiles of Leaves of Bilberry (*Vaccinium myrtillus* L.) and Lingonberry (*Vaccinium vitis-idaea* L.). J. Agric. Food Chem..

[36] Cyboran S., Oszmiański J., Kleszczyńska H. (2013). Modification of the Lipid Phase of Biological and Model Membranes by Bilberry Leaf Extract. Food Biophys..

[37] Takács I., Szekeres A., Takács Á., Rakk D., Mézes M., Polyák Á., Lakatos L., Gyémánt G., Csupor D., Kovács K.J. (2020). Wild Strawberry, Blackberry, and Blueberry Leaf Extracts Alleviate Starch-Induced Hyperglycemia in Prediabetic and Diabetic Mice. Planta Med..

[38] Toomik P., Püssa T., Raal A. (2014). Variability of Procyanidin Type A- and -B Trimers Content in Aerial Parts of Some *Vaccinium* Species and Cultivars. Nat. Prod. Commun..

[39] Martz F., Jaakola L., Julkunen-Tiitto R., Stark S. (2010). Phenolic Composition and Antioxidant Capacity of Bilberry (*Vaccinium myrtillus*) Leaves in Northern Europe Following Foliar Development and Along Environmental Gradients. J. Chem. Ecol..

[40] Luca S.V., Bujor A., Miron A., Aprotosoaie A.C., Skalicka-Woźniak K., Trifan A. (2020). Preparative Separation and Bioactivity of Oligomeric Proanthocyanidins. Phytochem. Rev..

[41] Nawrot-Hadzik I., Matkowski A., Hadzik J., Dobrowolska-Czopor B., Olchowy C., Dominiak M., Kubasiewicz-Ross P. (2021). Proanthocyanidins and Flavan-3-Ols in the Prevention and Treatment of Periodontitis—Antibacterial Effects. Nutrients.

[42] Hohtola A., Jaakola L., Määttä-Riihinen K., Kärenlampi S. (2004). Activation of Flavonoid Biosynthesis by Solar Radiation in Bilberry (*Vaccinium myrtillus* L.) Leaves. Planta.

[43] Vrancheva R., Ivanov I., Dincheva I., Badjakov I., Pavlov A. (2021). Triterpenoids and Other Non-Polar Compounds in Leaves of Wild and Cultivated Vaccinium Species. Plants.

[44] Szakiel A., Pączkowski C., Huttunen S. (2012). Triterpenoid Content of Berries and Leaves of Bilberry *Vaccinium myrtillus* from Finland and Poland. J. Agric. Food Chem..

[45] Castellano J.M., Ramos-Romero S., Perona J.S. (2022). Oleanolic Acid: Extraction, Characterization and Biological Activity. Nutrients.

[46] Bangladesh Journal of Botany Chemical Composition and Biological Activities of the Essential Oil from the Leaves of *Vaccinium myrtillus* L. https://www.banglajol.info/index.php/BJB/article/view/49098.

[47] Morkunas I., Woźniak A., Mai V.C., Rucińska-Sobkowiak R., Jeandet P. (2018). The Role of Heavy Metals in Plant Response to Biotic Stress. Molecules.

[48] Kandziora-Ciupa M., Gospodarek J., Nadgórska-Socha A. (2022). Pollution and Ecological Risk Assessment of Heavy Metals in Forest Soils with Changes in the Leaf Traits and Membrane Integrity of *Vaccinium myrtillus* L. Eur. J. For. Res..

[49] U.S. Food and Drug Administration (2020). Lead in Food, Foodwares, and Dietary Supplements.

[50] Amelia T., Bianca-Eugenia O., Amalia M. (2020). Substantele Minerale, Factori Esentiali in Nutritie. O Abordare Din Perspectiva Bio-Chimica Si Farmacologica.

[51] Nordløkken M., Berg T., Flaten T.P., Steinnes E. (2015). Essential and Non-Essential Elements in Natural Vegetation in Southern Norway: Contribution from Different Sources. Sci. Total Environ..

[52] Eeva T., Holmström H., Espín S., Sánchez-Virosta P., Klemola T. (2018). Leaves, Berries and Herbivorous Larvae of Bilberry *Vaccinium myrtillus* as Sources of Metals in Food Chains at a Cu-Ni Smelter Site. Chemosphere.

[53] Kandziora-Ciupa M., Ciepał R., Nadgórska-Socha A., Barczyk G. (2013). A Comparative Study of Heavy Metal Accumulation and Antioxidant Responses in *Vaccinium myrtillus* L. Leaves in Polluted and Non-Polluted Areas. Environ. Sci. Pollut. Res..

[54] Miljković V.M., Nikolić G.S., Zvezdanović J., Mihajlov-Krstev T., Arsić B.B., Miljković M.N. (2018). Phenolic Profile, Mineral Content and Antibacterial Activity of the Methanol Extract of *Vaccinium myrtillus* L. Not. Bot. Hortic. Agrobot..

[55] Khodavirdipour A., Haddadi F., Keshavarzi S. (2020). Chromium Supplementation; Negotiation with Diabetes Mellitus, Hyperlipidemia and Depression. J. Diabetes Metab. Disord..

[56] Kooshki F., Tutunchi H., Vajdi M., Karimi A., Niazkar H.R., Shoorei H., Pourghassem Gargari B. (2021). A Comprehensive Insight into the Effect of Chromium Supplementation on Oxidative Stress Indices in Diabetes Mellitus: A Systematic Review. Clin. Exp. Pharmacol. Physiol..

[57] Rothwell J.A., Urpi-Sarda M., Boto-Ordoñez M., Llorach R., Farran-Codina A., Barupal D.K., Neveu V., Manach C., Andres-Lacueva C., Scalbert A. (2016). Systematic Analysis of the Polyphenol Metabolome Using the Phenol-Explorer Database. Mol. Nutr. Food Res..

[58] Khan J., Deb P.K., Priya S., Medina K.D., Devi R., Walode S.G., Rudrapal M. (2021). Dietary Flavonoids: Cardioprotective Potential with Antioxidant Effects and Their Pharmacokinetic, Toxicological and Therapeutic Concerns. Molecules.

[59] Murota K., Nakamura Y., Uehara M. (2018). Flavonoid Metabolism: The Interaction of Metabolites and Gut Microbiota. Biosci. Biotechnol. Biochem..

[60] Hostetler G.L., Ralston R.A., Schwartz S.J. (2017). Flavones: Food Sources, Bioavailability, Metabolism, and Bioactivity. Adv. Nutr..

[61] Chen J., Yang J., Ma L., Li J., Shahzad N., Kim C.K. (2020). Structure-Antioxidant Activity Relationship of Methoxy, Phenolic Hydroxyl, and Carboxylic Acid Groups of Phenolic Acids. Sci. Rep..

[62] Chen L., Teng H., Xie Z., Cao H., Cheang W.S., Skalicka-Woniak K., Georgiev M.I., Xiao J. (2018). Modifications of Dietary Flavonoids towards Improved Bioactivity: An Update on Structure–Activity Relationship. Crit. Rev. Food Sci. Nutr..

[63] Jîtcă G., Ősz B.E., Tero-Vescan A., Miklos A.P., Rusz C.-M., Bătrînu M.-G., Vari C.E. (2022). Positive Aspects of Oxidative Stress at Different Levels of the Human Body: A Review. Antioxidants.

[64] Olszowy M. (2019). What Is Responsible for Antioxidant Properties of Polyphenolic Compounds from Plants?. Plant Physiol. Biochem..

[65] Tian Y., Puganen A., Alakomi H.-L., Uusitupa A., Saarela M., Yang B. (2018). Antioxidative and Antibacterial Activities of Aqueous Ethanol Extracts of Berries, Leaves, and Branches of Berry Plants. Food Res. Int..

[66] Cheesman M.J., Ilanko A., Blonk B., Cock I.E. (2017). Developing New Antimicrobial Therapies: Are Synergistic Combinations of Plant Extracts/Compounds with Conventional Antibiotics the Solution?. Pharmacogn. Rev..

[67] Breijyeh Z., Jubeh B., Karaman R. (2020). Resistance of Gram-Negative Bacteria to Current Antibacterial Agents and Approaches to Resolve It. Molecules.

[68] Rotaru L.T. (2019). Correlation of the Theoretical Study with the Experimental Determination of the Antibacterial Effect of *Vaccinium myrtillus* Folium (VM-f) Plant Extract. Rev. Chim..

[69] Rice L.B. (2008). Federal Funding for the Study of Antimicrobial Resistance in Nosocomial Pathogens: No ESKAPE. J. Infect. Dis..

[70] Dhakal A., Sbar E. (2022). Aflatoxin Toxicity. StatPearls.

[71] Vamvakas S.-S., Chroni M., Genneos F., Gizeli S. (2021). *Vaccinium myrtillus* L. Dry Leaf Aqueous Extracts Suppress Aflatoxins Biosynthesis by Aspergillus Flavus. Food Biosci..

[72] Gregg E.W., Sattar N., Ali M.K. (2016). The Changing Face of Diabetes Complications. Lancet Diabetes Endocrinol..

[73] Bahadoran Z., Mirmiran P., Azizi F. (2013). Dietary Polyphenols as Potential Nutraceuticals in Management of Diabetes: A Review. J. Diabetes Metab. Disord..

[74] Dirir A.M., Daou M., Yousef A.F., Yousef L.F. (2022). A Review of Alpha-Glucosidase Inhibitors from Plants as Potential Candidates for the Treatment of Type-2 Diabetes. Phytochem. Rev..

[75] Samarakoon D.N.A.W., Uluwaduge D.I., Siriwardhene M.A. (2020). Mechanisms of Action of Sri Lankan Herbal Medicines Used in the Treatment of Diabetes: A Review. J. Integr. Med..

[76] Sales P.M., Souza P.M., Simeoni L.A., Magalhães P.O., Silveira D. (2012). α-Amylase Inhibitors: A Review of Raw Material and Isolated Compounds from Plant Source. J. Pharm. Pharm. Sci..

[77] Fraga-Corral M., Otero P., Echave J., Garcia-Oliveira P., Carpena M., Jarboui A., Nuñez-Estevez B., Simal-Gandara J., Prieto M.A. (2021). By-Products of Agri-Food Industry as Tannin-Rich Sources: A Review of Tannins’ Biological Activities and Their Potential for Valorization. Foods.

[78] Márquez Campos E., Jakobs L., Simon M.-C. (2020). Antidiabetic Effects of Flavan-3-Ols and Their Microbial Metabolites. Nutrients.

[79] Sidorova Y., Shipelin V., Mazo V., Zorin S., Petrov N., Kochetkova A. (2017). Hypoglycemic and Hypolipidemic Effect of *Vaccinium myrtillus* L. Leaf and *Phaseolus vulgaris* L. Seed Coat Extracts in Diabetic Rats. Nutrition.

[80] Ştefănescu (Braic) R., Vari C., Imre S., Huţanu A., Fogarasi E., Todea T., Groşan A., Eşianu S., Laczkó-Zöld E., Dogaru M. (2018). *Vaccinium* Extracts as Modulators in Experimental Type 1 Diabetes. J. Med. Food.

[81] Zhao Z., Chen Y., Li X., Zhu L., Wang X., Li L., Sun H., Han X., Li J. (2022). Myricetin Relieves the Symptoms of Type 2 Diabetes Mice and Regulates Intestinal Microflora. Biomed. Pharmacother..

[82] Dhanya R. (2022). Quercetin for Managing Type 2 Diabetes and Its Complications, an Insight into Multitarget Therapy. Biomed. Pharmacother..

[83] Shi G.-J., Li Y., Cao Q.-H., Wu H.-X., Tang X.-Y., Gao X.-H., Yu J.-Q., Chen Z., Yang Y. (2019). In Vitro and in Vivo Evidence That Quercetin Protects against Diabetes and Its Complications: A Systematic Review of the Literature. Biomed. Pharmacother..

[84] Ong K.W., Hsu A., Tan B.K.H. (2013). Anti-Diabetic and Anti-Lipidemic Effects of Chlorogenic Acid Are Mediated by Ampk Activation. Biochem. Pharmacol..

[85] Alim Z., Kilinç N., Şengül B., Beydemir Ş. (2017). Inhibition Behaviours of Some Phenolic Acids on Rat Kidney Aldose Reductase Enzyme: An in Vitro Study. J. Enzyme Inhib. Med. Chem..

[86] Ștefanescu R., Fülöp E., Demian L., Vari C., Ősz B.-E., Groșan A., Laczko-Zöld E., Chibelean B. (2022). Efficacy of Natural Polyphenolic Compounds from Bilberry and Blueberry on The Metabolic Alterations Induced By Streptozotocin In Rats. Farmacia.

[87] Dostalek M., Akhlaghi F., Puzanovova M. (2012). Effect of Diabetes Mellitus on Pharmacokinetic and Pharmacodynamic Properties of Drugs. Clin. Pharmacokinet..

[88] Srinivas N.R. (2015). Strategies for Preclinical Pharmacokinetic Investigation in Streptozotocin-Induced Diabetes Mellitus (DMIS) and Alloxan-Induced Diabetes Mellitus (DMIA) Rat Models: Case Studies and Perspectives. Eur. J. Drug Metab. Pharmacokinet..

[89] Stoica M.C., Vari C.E., Imre S., Vancea S., Dogaru M.T., Cara E., Tar D. (2015). Correlations Between the Stages of Kidney Disease and the Pharmacokinetic Parameters of Orally Administered Ciprofloxacin at Patients with Chronic Kidney Disease. Farmacia.

[90] Popova A., Mihaylova D. (2019). Antinutrients in Plant-Based Foods: A Review. Open Biotechnol. J..

[91] Chehri A., Yarani R., Yousefi Z., Shakouri S.K., Ostadrahimi A., Mobasseri M., Araj-Khodaei M. (2022). Phytochemical and Pharmacological Anti-Diabetic Properties of Bilberries (*Vaccinium myrtillus*), Recommendations for Future Studies. Prim. Care Diabetes.

[92] Tundis R., Tenuta M.C., Loizzo M.R., Bonesi M., Finetti F., Trabalzini L., Deguin B. (2021). *Vaccinium* Species (Ericaceae): From Chemical Composition to Bio-Functional Activities. Appl. Sci..

